# Explicit and implicit motor simulations are impaired in individuals with aphantasia

**DOI:** 10.1093/braincomms/fcae072

**Published:** 2024-03-21

**Authors:** William Dupont, Charalambos Papaxanthis, Carol Madden-Lombardi, Florent Lebon

**Affiliations:** UFR des Sciences du Sport, INSERM UMR1093-CAPS, Université Bourgogne, Dijon F-21000, France; UFR des Sciences du Sport, INSERM UMR1093-CAPS, Université Bourgogne, Dijon F-21000, France; UFR des Sciences du Sport, INSERM UMR1093-CAPS, Université Bourgogne, Dijon F-21000, France; Centre National de la Recherche Scientifique (CNRS), Paris, France; UFR des Sciences du Sport, INSERM UMR1093-CAPS, Université Bourgogne, Dijon F-21000, France; Institut Universitaire de France (IUF), Paris, France

**Keywords:** motor imagery, action observation, aphantasia, mental representation

## Abstract

Individuals with aphantasia report having difficulties or an inability to generate visual images of objects or events. So far, there is no evidence showing that this condition also impacts the motor system and the generation of motor simulations. We probed the neurophysiological marker of aphantasia during explicit and implicit forms of motor simulation, i.e. motor imagery and action observation, respectively. We tested a group of individuals without any reported imagery deficits (phantasics) as well as a group of individuals self-reporting the inability to mentally simulate images or movements (aphantasics). We instructed the participants to explicitly imagine a maximal pinch movement in the visual and kinaesthetic modalities and to observe a video showing a pinch movement. By means of transcranial magnetic stimulation, we triggered motor-evoked potentials in the target right index finger. As expected, the amplitude of motor-evoked potentials, a marker of corticospinal excitability, increased for phantasics during kinaesthetic motor imagery and action observation relative to rest but not during visual motor imagery. Interestingly, the amplitude of motor-evoked potentials did not increase in any of the conditions for the group of aphantasics. This result provides neurophysiological evidence that individuals living with aphantasia have a real deficit in activating the motor system during motor simulations.

## Teaser

Dupont *et al*. studied aphantasic individuals who were unable or struggled to generate mental visual images. They questioned whether this inability extended to motor processes, concluding that there was no increase of corticospinal excitability typically measured while imagining or observing an action. This supports an alteration of motor simulation processes.

## Graphical Abstract

**Graphical Abstract:**
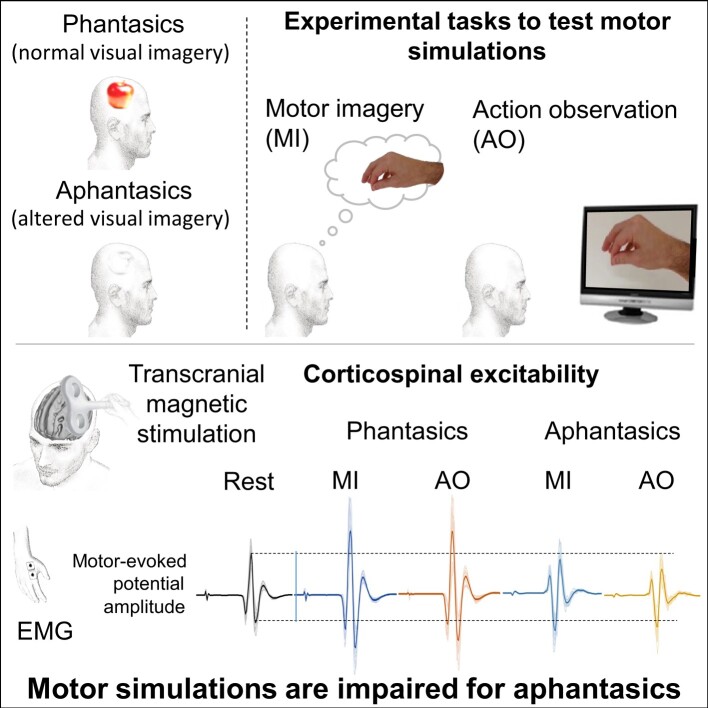

See Esselaar, Holmes, Scott and Wright (https://doi.org/10.1093/braincomms/fcae084) for a scientific commentary on this article.

## Introduction

The generation of mental simulations is a fundamental characteristic of human existence, allowing us to retrieve and/or predict the sensorimotor consequences of an action (motor imagery) or to understand the actions of others (action observation). These internal simulations mirror our real experience and are therefore multimodal, encompassing mental imagery (e.g. visual, auditory or olfactory imagery and mental rotation) but also extend to motor-related aspects such as motor imagery and action observation. Indeed, motor imagery and action observation are identified as explicit and implicit motor simulations, respectively,^1^ both activating the sensorimotor system, although no movement is produced.^2-8^ Such mental processes are relevant interventions to improve motor learning and to promote motor rehabilitation.^7,9-11^

Although the generation and use of mental simulation seems a natural process, a small portion of the population (∼4%^12^), called aphantasics, reports being unable or struggling to create mental images of an event.^13-16^ Behaviourally, this deficit is mainly evaluated by subjective reports of visual imagery vividness.^13-18^ Recently, Kay and colleagues used physiological methodologies, such as binocular rivalry and eye tracking, to demonstrate a lack of content-specific response in aphantasics (despite their engagement in the task), indicating a difficulty for visualizing shapes.^19,20^ However, the available psychological and physiological evidence at present remains insufficient to determine whether aphantasics are actually unable to generate mental simulations or whether such difficulty is a matter of strategy or metacognition. In addition, it remains unclear whether this deficit that is typically measured in visual imagery would also affect motor imagery.

The present paper aims to shed new light on aphantasics’ condition by exploring their ability to activate the motor system while engaging in an explicit form of motor simulation, i.e. motor imagery, using neurological measures that do not rely on self-report. It is, however, possible that any observed psychophysical or neurophysiological deficits could be related to individual differences in strategies or motivation (‘I don’t think I can imagine, so I don’t try’). Therefore, we also tested the modulation of the motor system during action observation, which engages an implicit form of motor simulation, unrelated to the participant’s efforts or strategies. For each of these simulation types, we measured corticospinal excitability by means of transcranial magnetic stimulation (TMS) as well as subjective reports of imagery vividness and utilization of imagery in everyday life. There is strong evidence in the literature that neurophysiological manifestations of motor simulations in individuals with normal imagery ability (i.e. phantasics) include the increase of corticospinal excitability in TMS studies during both motor imagery^7,21-25^ and action observation.^26-33^

Our main hypothesis is straightforward: if aphantasics are not able to create motor simulations (self-reports), we would not observe an increase of corticospinal excitability during motor imagery and action observation that is typically measured in phantasics. The absence of facilitation in corticospinal excitability during motor simulations would be a relevant marker of aphantasia.

## Materials and methods

### Participants

Thirty-four right-handed aphantasic (*n* = 17) and phantasic (*n* = 17) participants were included in this cross-sectional study. The recruitment process employed a mailing list at the University of Bourgogne and the extensive network of an aphantasics association in Dijon. Potential aphantasics were asked to contact us if they scored 23 or below on the French version of the Vividness of Visual Imagery Questionnaire^34^ (17.23 ± 2.75; range of scores: 16–23), which was included in the recruitment mail and self-administered at home. Aphantasia (and phantasia) was then confirmed in our lab with the visual modality of the Vividness of Movement Imagery Questionnaire, second version (VMIQ-2).^35^ Six participants were excluded from the analysis (see Statistical analysis for details). We analysed the data of 14 aphantasics (8 women: mean age, 21; range, 18–26) and 14 phantasics (5 women: mean age, 23; range, 19–26). We confirmed right laterality with the Edinburgh inventory,^36^ and each participant provided us with their Autism Spectrum Quotient score,^37^ which was self-administered at home. All participants were French native speakers. They completed the questionnaire by Lefaucheur *et al*.^38^ for TMS eligibility, and they provided written consent to confirm their participation. All procedures (excluding preregistration) were approved by an ethics committee (CPP SOOM III, ClinicalTrials.gov Identifier: NCT03334526) and were in accordance with the Declaration of Helsinki.

### Procedure and stimuli

The current study included two experimental sessions, 1 week apart. In the first session, we assessed imagery ability with self-report questionnaires (behavioural session). In the second session, we measured corticospinal excitability by means of single-pulse TMS delivered at rest, during visual and kinaesthetic motor imagery and during action observation (neurophysiological session).

### Behavioural session

All participants completed the VMIQ-2^35^ and the Spontaneous Use of Imagery Scale (SUIS).^39^ In the VMIQ-2, participants used three modalities (external visual imagery, internal visual imagery, kinaesthetic imagery) to imagine several actions. After each imagined movement, they determined how vivid their imagery was by means of a 1–5 Likert scale (with 1, ‘Perfectly clear and vivid as normal vision’, and 5, ‘No image at all, you only think about the movement’). In the SUIS questionnaire, participants rated on a five-point scale their general tendency to use visual mental imagery in everyday situations (from 1, ‘never appropriate’, to 5, ‘always completely appropriate’).

### Neurophysiological session

In this session, we used TMS to probe corticospinal excitability at rest, during visual and kinaesthetic motor imagery and during action observation. Participants sat in front of a 19 inch LCD monitor, and we utilized a custom-designed software to deliver precisely timed TMS pulses. First, we delivered sixteen TMS pulses at rest (fixation cross), which served as baseline.

**Figure 1:**
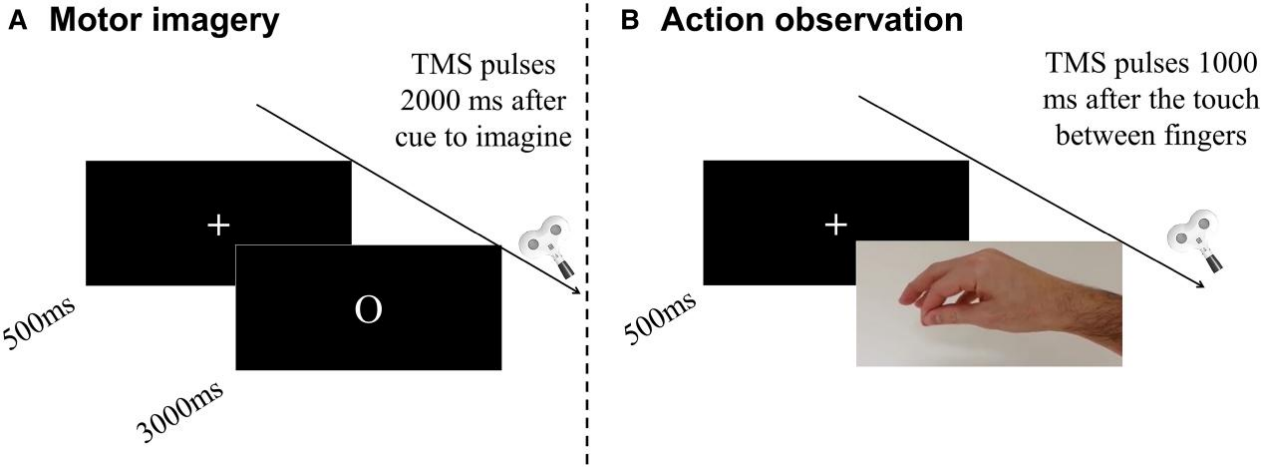
**Experimental procedure.** Each trial started with a fixation cross to indicate the beginning of a trial. (**A**) Motor imagery: the participants imagined holding a pinch between the index finger and the thumb of the right hand. We delivered TMS pulses 2000 ms after the appearance of the imagery onset (‘O’). (**B**) Action observation: the participants observed a video of pinch movements. We delivered TMS pulses 1000 ms after the touch between the index finger and the thumb.

In each motor imagery block, participants were instructed to imagine 16 maximum voluntary contractions of pinch movements held for 3 s, either in a first-person visual or kinaesthetic modality, corresponding to the experimental conditions visual imagery and kinaesthetic imagery, respectively. The following instructions were provided: ‘try to imagine yourself performing the pinch movement, by visualizing the movement just as if you were watching your fingers move (for visual imagery) or feeling the fingers’ sensations as if you were doing the movement (for kinaesthetic imagery)’. TMS pulses were delivered 2000 ms after the cue to imagine (‘O’ on the screen). The intertrial interval was 7000 ms. In the action observation block, participants were instructed to observe a video of a first-person pinch movement. For each of the 16 trials, a TMS pulse was delivered 1000 ms after the touch between the index and the thumb fingers (Fig. 1). The blocks were counterbalanced between participants. The selection of 16 TMS pulses per condition represented a good compromise between the duration of the experiment, which included 5 distinct conditions (2 rest blocks, 2 motor imagery tasks and 1 observation task), and a high probability to reach 95% confidence interval of reliable estimate for corticospinal excitability.^40^

We placed 10 mm diameter surface electrodes (Contrôle Graphique Médical, Brice Comte-Robert, France) over the right first dorsal interosseous (FDI) muscle to record surface electromyography (EMG). We shaved and cleaned the skin before positioning the electrodes to reduce EMG signal noise (<20 μV). We amplified EMG signal with a bandpass filter (10–500 Hz) and digitized it at 2000 Hz (AcqKnowledge; Biopac Systems, Inc., Goleta, CA). We calculated the root mean square EMG signal (EMGrms) preceding stimulation to guarantee that amplitudes of motor-evoked potentials (MEP) in experimental conditions were not contaminated by muscle activation.

We delivered single-pulse TMS over the right FDI motor area by means of a figure-eight coil (70 mm diameter) connected to a Magstim 200 stimulator (Magstim Company Ltd, Whitland). We placed the TMS coil tangentially to the scalp with the handle pointing backward and laterally at a 45° angle from the midline. We used a neuronavigation system (Brainsight, Rogue Research Inc.), with an ‘approach similar to probabilistic without individual MRI’,^41^ to identify the muscle ‘hotspot’, i.e. the position where stimulation evoked the highest and most consistent MEP amplitude for the FDI muscle. We determined this position with a regular grid of 4 × 4 coil positions with a spacing of 1 cm (centred above the FDI cortical area *x* = −37, *y* = −19, *z* = 63^42,43^). Then, we estimated the resting motor threshold for each participant, i.e. the minimal TMS intensity required to evoke MEPs of 50 µV peak-to-peak amplitude in the right FDI muscle for 5 out of 10 trials.^44^ During the experimental conditions, we set the intensity of TMS pulses to 130% of the resting motor threshold.

### Statistical analysis

Using G* Power (version 3.1.9.4.^45^), we estimated that 15 participants per group would be needed, based on a large effect size of 1 and a power of 0.8. Due to potential loss of data (10%), we recruited 34 participants in total.

We used Matlab (The MathWorks, Natick, Massachusetts, USA) to extract EMG, and we measured the peak-to-peak MEP amplitude. Before statistical analysis, we discarded MEPs outside the range of ±2 SDs from individual means for each condition (3.75% of all data). We normalized the average MEP amplitude for each condition to rest. Six participants (three phantasics and three aphantasics) were removed from the final analysis due to extreme values for MEP amplitude or VMIQ scores (outside the range of 2 SDs). Using Shapiro–Wilk and Mauchly tests, we checked the normality and sphericity of the data. One-sided *t*-tests were used to compare MEPs between each group during kinaesthetic and visual imagery and action observation. Moreover, one-sample *t*-tests were used to compare condition MEPs with zero (rest). The associated Bayes factor analyses were conducted to evaluate the presence or absence of an effect. Finally, we used a Friedmann ANOVA to compare the EMGrms before the stimulation artefact in all our conditions for each group (see Supplementary Table 1) to ensure that MEPs were not biased by muscle activation preceding stimulation. We performed data analysis and statistics with Statistica software (Stat Soft, France). We present the data as mean values (±SD) and we set the alpha value at 0.05.

## Results

### Explicit motor simulation (motor imagery)

**Figure 2:**
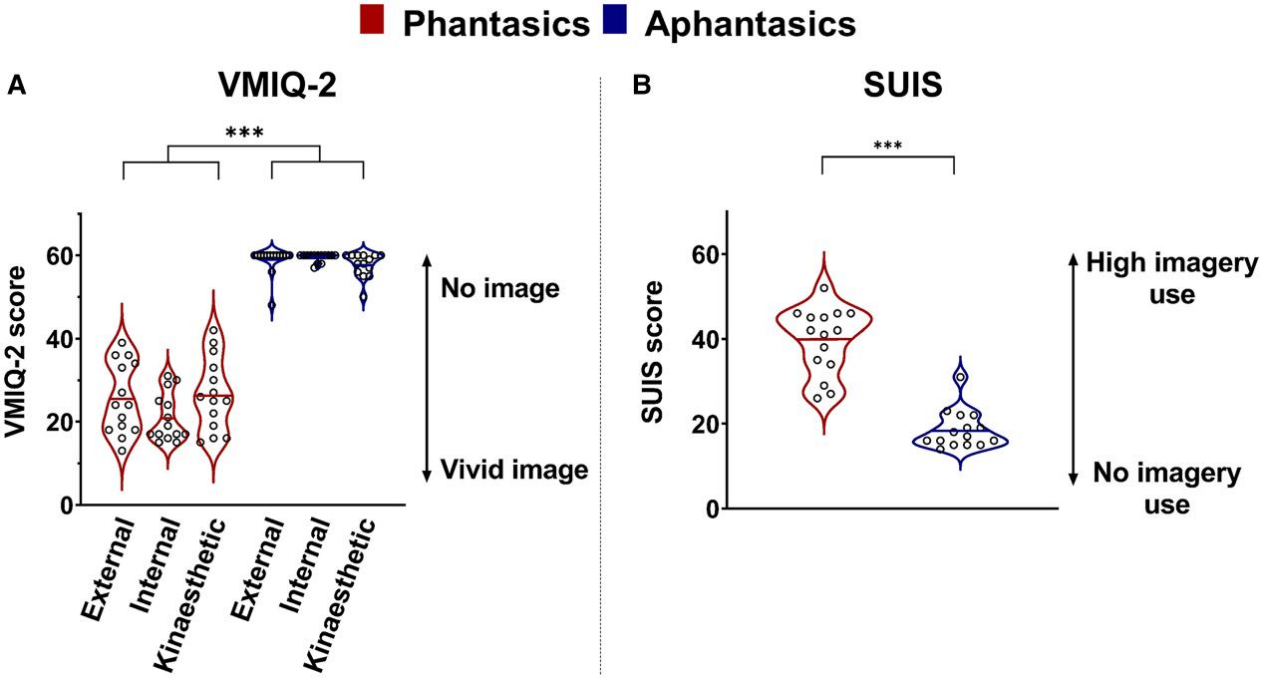
**Self-report questionnaires.** Violin plots represent scores on (**A**) the Vividness of Movement Imagery Questionnaire-2 (VMIQ-2) and (**B**) the SUIS questionnaire for phantasics and aphantasics. Thick horizontal lines mark the mean, and circles represent individual data. For VMIQ-2, lowest (12) and highest (60) scores represent the highest and lowest vividness, respectively. The participants reported the vividness of imagined movements for three modalities (external visual, internal visual and kinaesthetic). The scale is inverted for the SUIS, where the lowest (12) and highest (60) scores represent lowest and highest use of mental imagery in everyday situations, respectively. A repeated measures ANOVA and an independent *t*-test were used for VMIQ-2 and SUIS, respectively. ***P < 0.001.

The average scores on self-report questionnaires confirm that individuals in the Aphantasic group (*n* = 14) had difficulty or were unable to explicitly create mental images of common actions, in comparison with individuals in the Phantasic group (*n* = 14). The main results and statistics of the Vividness of Movement Imagery Questionnaire-2^35^ and the SUIS^39^ are presented in Fig. 2 (and in Supplementary Table 2 for details). These subjective reports are in line with the literature focusing on the creation of mental visual images.^13,16,20^

**Figure 3:**
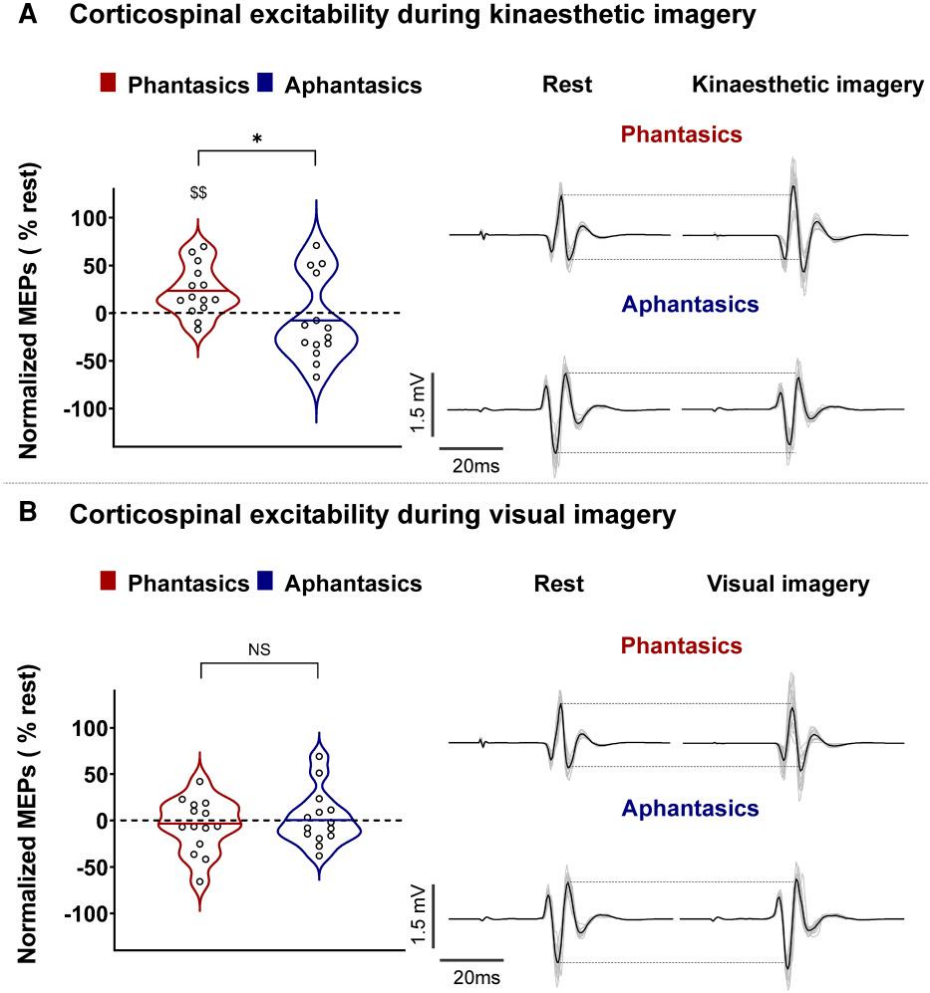
**Corticospinal excitability during kinaesthetic and visual imagery for phantasics and aphantasics.** Violin plots on the left side represent normalized MEPs during kinaesthetic (**A**) and visual imagery (**B**). Thick horizontal lines mark the mean. Circles represent individual data. The right side of the panel illustrates raw MEPs of a typical subject in shaded lines, and the dark line is the average MEP of the condition for this participant. Independent *t*-tests were used for intergroup comparisons. *P < 0.05 indicates a significant difference between the two groups, and $$ = *P* < 0.01 indicates a significant difference from zero (rest).

These subjective reports were supplemented by neurophysiological data. As expected, corticospinal excitability increased during kinaesthetic but not visual motor imagery in phantasics,^46^ whereas it was not modulated in either imagery modality for aphantasics (Fig. 3). More specifically, MEP amplitude increased during kinaesthetic imagery in comparison with rest for phantasics (23.43 ± 26.39%; *t* = 3.321, *P* = 0.006, Bayes factor_10_ = 9.281), but not for aphantasics (−7.45 ± 43.49%; *t* = −0.640, *P* = 0.533, Bayes factor_10_ = 0.323). The percentage of MEP amplitude increase differed between the two groups (one-sided independent *t*-test: *t*(_26_) = 2.271, *P* = 0.016; Cohen’s *d* = 0.89, Bayes factor_10_ = 4.296). During visual imagery, neither group increased MEP amplitude in comparison with rest (−5.57 ± 28.92%, *t* = −0.721, *P* = 0.484, Bayes factor_10_ = 0.338, and 2.35 ± 29.45%, *t* = 0.298, *P* = 0.770, Bayes factor_10_ = 0.281 for phantasics and aphantasics, respectively). The percentage of MEP amplitude increase was not statistically different between the two groups (one-sided independent *t*-test: *t*(_26_) = −0.718, *P* = 0.240; Cohen’s *d* = 0.28, Bayes factor_10_ = 0.232). The Bayes factor analysis not only provides moderate support for the presence of an effect of kinaesthetic motor imagery in phantasics but importantly provides evidence for the absence of such an effect in aphantasics. This finding underscores the significance of our results in distinguishing these two groups of participants and their respective corticospinal excitability modulations.

### Implicit motor simulation (action observation)

**Figure 4:**
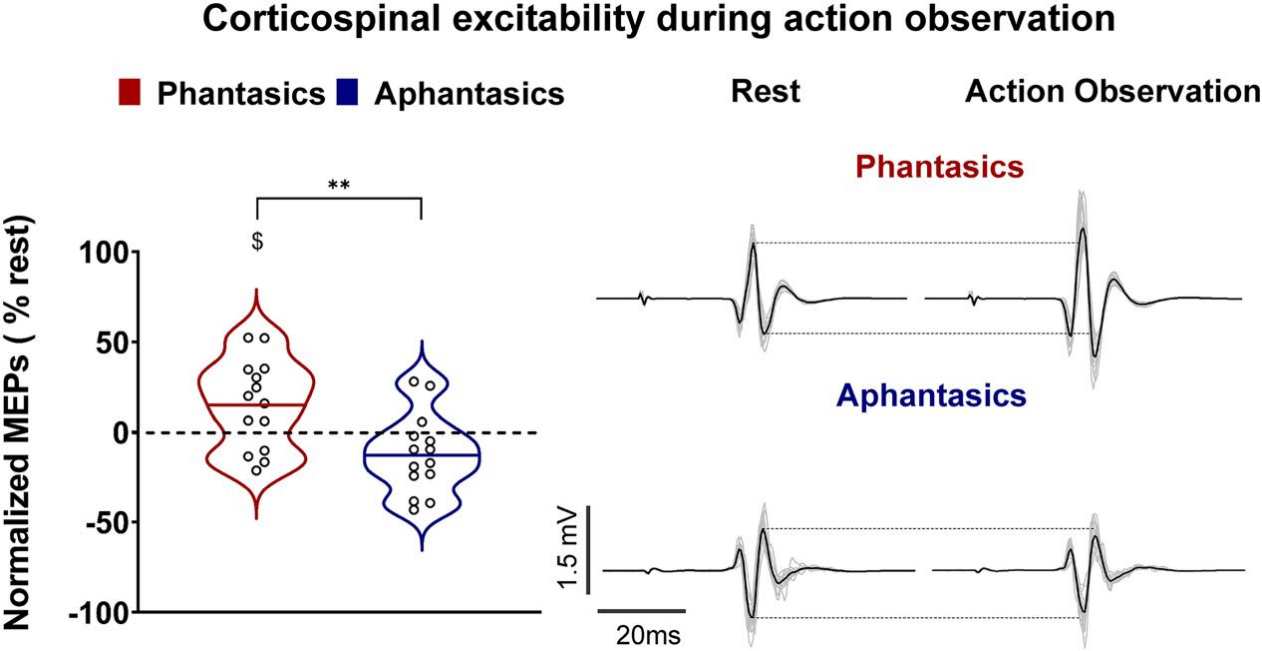
**Corticospinal excitability during action observation for phantasics and aphantasics.** Violin plots on the left side represent normalized MEPs during action observation. Thick horizontal lines mark the mean. Circles represent individual data. The right side of the panel illustrates raw MEPs of a typical subject in shaded lines, and the dark line is the average MEP of the condition for this participant. Independent *t*-tests were used for intergroup comparisons. **P < 0.005 indicates a significant difference between the two groups, and $ = *P* < 0.05 indicates a significant difference from zero (rest).

While the previous kinaesthetic imagery measure provides strong evidence that aphantasics do not explicitly generate motor images when prompted, it remains possible that they are capable of simulating, but avoid doing so, perhaps due to their impressions of difficulty or failure. Therefore, we also measured corticospinal excitability during an implicit form of motor simulation that is less influenced by participants’ efforts and strategies, i.e. action observation. As expected, corticospinal excitability increased during action observation in comparison with rest for phantasics (15.34 ± 24.52%, *t* = 2.342, *P* = 0.036, Bayes factor_10_ = 2.042) but was unchanged for aphantasics (−12.34 ± 21.96%, *t* = −2.102, *P* = 0.056, Bayes factor_10_ = 1.452). The percentage of MEP amplitude increase was statistically different between the two groups (one-sided independent *t*-test: *t*(_26_) = 3.148, *P* = 0.002; Cohen’s *d* = 1.23, Bayes factor_10_ = 20.031; Fig. 4). While the Bayes factor analyses only provide weak evidence for the existence of effects of action observation in phantasics and aphantasics, there is indeed strong evidence for a significant difference between these two groups. Nevertheless, Autism Spectrum Quotients did not differ between our two groups (3.21 ± 2.25 and 4.71 ± 2.49 for phantasics and aphantasics, respectively; *t* = 1.67, *P* = 0.10, Cohen’s *d* = 0.66), thereby suggesting that these scores cannot account for the differences in corticospinal excitability modulations observed during action observation between the two groups.

## Discussion

The present study provides several notable findings related to explicit and implicit motor simulation and aphantasia. First, the increase in corticospinal excitability during explicit motor imagery for phantasics but not aphantasics demonstrates that only those able to generate mental simulations of hand actions showed motor cortex engagement. Second, this same effect during action observation, an implicit form of motor simulation, demonstrates once again that aphantasia entails a real impairment outside the control of individuals (strategies, volition, metacognition).

Concerning the first point, the self-reported estimates of vividness (VMIQ-2) and utilization of imagery in everyday life (SUIS) suggest that participants living with aphantasia are less able (or unable) to simulate movements, corroborating previous research on mental imagery.^13,16,20^ However, it remains unclear whether such reports reflect a real neurophysiological impairment or merely a metacognitive deficit. Thus, we used the modulation of corticospinal excitability as a marker of the generation of motor simulation. We confirmed the increase of corticospinal excitability during kinaesthetic imagery in phantasics^7,21-23,25,47^ and the absence of such an increase in the motor system during visual imagery.^46^ More interestingly, we found that aphantasics did not exhibit any increase of corticospinal excitability during imagery in either modality. These psychological and physiological findings support the view that aphantasia entails a real impairment in the generation of motor images, rather than a metacognition failure.^19,20,48^ To note, our sample of aphantasics was self-selected (volunteers who identified themselves as unable to generate imagery), and our phantasic sample was not prescreened for imagery ability. Therefore, our groups may not reflect the maximal difference between phantasic and aphantasic individuals. Specifically, some of our aphantasics may not be fully impaired in their simulation ability but rather possess only a limited ability to simulate.^15^ Our data also lend support to the idea that certain aphantasics can present complete impairments in one modality (e.g. visual with self-report questionnaires) while exhibiting at least limited abilities to simulate in another modality (e.g. kinaesthetic with self-report questionnaires and TMS data).^13^

Concerning the second point, the current study on action observation supports the recent findings of our team showing that aphantasia also encompasses an inability to engage in action reading,^49^ another form of implicit motor simulation. This result also extends recent investigations demonstrating longer reaction times during mental rotation^18,50,51^ (but see also Milton *et al*.^14^) and involuntary simulations during night-time dreams.^13,16,18,52^ As already described for motor imagery, we found that action observation increased corticospinal excitability in phantasics but not aphantasics, when compared with rest. Therefore, aphantasics have difficulties, if not an inability, to generate explicit and implicit motor simulations.

Although Autism Spectrum Quotients did not differ between our two groups, it is important to acknowledge that previous research has suggested a higher presence of autistic traits in aphantasics than phantasics,^37^ which could have an influence on imagery ability or MEP amplitudes during action observation.^53^ It is also important to acknowledge that we did not assess task motivation, which could have an influence on motor imagery and action observation. In addition, as recruiting aphantasic participants presents significant challenges, we were unable to ensure an equal distribution of male and female participants within this group and between the two groups. However, gender does not seem to be a confounding factor as no effect was observed for this group in an ANOVA with gender as a categorical factor and corticospinal excitability increase during motor imagery as the dependent variable (*F*(1,26) = 0.002, *P* = 0.968, ηp² < 0.001). Moreover, to our knowledge, no study has reported a gender-specific effect on corticospinal excitability during motor imagery.

In conclusion, our neurophysiological data support the idea that aphantasia is marked by a measurable lack of activation in the motor system, even when motor simulation should be engaged implicitly rather than explicitly. The modulation of MEP amplitude during explicit and implicit motor simulation may be a relevant tool to characterize aphantasia in the motor domain.

## Supplementary Material

fcae072_Supplementary_Data

## Data Availability

All data from this study are available at https://osf.io/4apxw/? view_only=f3dd901d53424462beaa8926cbda6dcc.
