# Supplementary material for: Explicit and implicit motor simulations are impaired in individuals with aphantasia
Source: Brain Commun. 2024 Mar 21;6(2):fcae072. doi: 10.1093/braincomms/fcae072 (PMC10957132; doi:10.1093/braincomms/fcae072)
Supplement: fcae072_Supplementary_Data [file fcae072_supplementary_data.docx]

**Supplementary Materials**

Supplementary Table 1: EMGrms activity (mean ±SD) in µV recorded for the first dorsal interosseous before the TMS artifact for each condition (window of 100ms prior the artifact). The absence of muscular pre-activity during action reading and motor imagery was confirmed by a Friedman Anova revealing no significant difference in EMGrms before the TMS artefact between rest, Visual Imagery, Kinaesthetic Imagery and Action Observation conditions for Aphantasic (uncorrected p=0.526; r=-0.019) and Phantasic individuals (uncorrected p=0.240; r=0.030).

|  | Rest | Action observation | Visual Imagery | Kinaesthetic Imagery |
| --- | --- | --- | --- | --- |
| Phantasics | 1.731  ±1.930 | 1.048  ±0.267 | 0.964  ±0.133 | 1.728  ±1.637 |
| Aphantasics | 1.544  ±1.186 | 1.186  ±0.366 | 1.462  ±0.588 | 1.475  ±0.811 |

Supplementary Table 2: Average scores on the Vividness of Movement Imagery Questionnaire-2 (VMIQ-2, Roberts et al., 2008) and the Spontaneous Use of Imagery Scale (SUIS, Ceschi and Pictet, 2018) for aphantasic and phantasic participants. The worst and best score for each modality at the VMIQ-2 is 60 and 12, respectively. The worst and best score for the SUIS questionnaire is 12 and 60, respectively. A repeated-measures ANOVA on VMIQ-2 scores revealed a main effect of Group (F_1,26_=404.97, p<0.001, ηp²=0.939), with greater imagery ability for Phantasic (24.36 ±6.18; Cohen’s d=7.89) than Aphantasic participants (58.69 ±1.61). We observed an interaction between Group and Perspective (F_2,52_=4.989, p=0.010, ηp²=0.160), demonstrating a significant difference between the internal (20.93 ±5.78) and kinaesthetic (26.57 ±8.75, p=0.021, Cohen's d=0.66) vividness of visual motor imagery in phantasics, and no difference between perspectives in aphantasics. Moreover, an independent T-test on SUIS scores yielded a greater utilization of imagery in everyday life for Phantasic individuals (39.71 ±8.12) than that for Aphantasic participants (18.50 ±4.64; t(_26_)=8.49, p<0.001, Cohen’s d=3.33).

|  | Questionnaires | | | |
| --- | --- | --- | --- | --- |
|  | VMIQ-2 | | | SUIS |
|  | External visual | Internal visual | Kinaesthetic |  |
| Phantasics | 25.57  ±8.59 | 20.93  ±5.78 | 26.57  ±8.75 | 39.71  ±8.12 |
| Aphantasics | 58.86  ±3.30 | 59.50  ±1.02 | 57.71  ±2.92 | 18.50  ±4.64 |

**References**

Ceschi G, Pictet A, eds. Imagerie mentale et psychothérapie. Mardaga; 2018

Roberts R, Callow N, Hardy L, Markland D, Bringer J. Movement imagery ability: Development and assessment of a revised version of the vividness of movement imagery questionnaire. Journal of Sport and Exercise Psychology. 2008;30(2):200-221. doi:10.1123/jsep.30.2.200
